# Bacteriophage cocktail supplementation improves growth performance, gut microbiome and production traits in broiler chickens

**DOI:** 10.1186/s40104-021-00570-6

**Published:** 2021-04-16

**Authors:** Santi Devi Upadhaya, Je Min Ahn, Jae Hyoung Cho, Jin Young Kim, Dae Kyung Kang, Sung Woo Kim, Hyeun Bum Kim, In Ho Kim

**Affiliations:** 1 Department of Animal Resource and Science, Dankook University, No.29 Anseodong, Cheonan, Choongnam 31116 South Korea; 2 Department of Animal Science, North Carolina State University, 116 Polk Hall, Box 7621, 120 W Broughton Dr, Raleigh, NC 27695 USA

**Keywords:** Bacteriophage, Broiler chickens, Gut microbiome, Performance

## Abstract

**Background:**

Effective antibiotic alternatives are urgently needed in the poultry industry to control disease outbreaks. Phage therapy mainly utilizes lytic phages to kill their respective bacterial hosts and can be an attractive solution to combating the emergence of antibiotic resistance in livestock.

**Methods:**

Five hundred and four, one-day-old broilers (Ross 308) were allotted to 1 of 4 treatment groups in a completely randomized design. Treatments consisted of CON (basal diet), PC (CON + 0.025% Avilamax®), BP 0.05 (CON + 0.05% bacteriophage), and BP 0.10 (CON + 0.10% bacteriophage).

**Results:**

A significant linear effect on body weight gain (BWG) was observed during days 1–7, days 22–35, and cumulatively in bacteriophage (BP) supplemented groups. The BWG tended to be higher (*P* = 0.08) and the feed intake (FI) was increased (*P* = 0.017) in the PC group over CON group. A greater (*P* = 0.016) BWG and trends in increased FI (*P* = 0.06) were observed in the experiment in birds fed PC than CON diet. At the genus level, the relative abundance of *Lactobacillus* was decreased in PC (65.28%), while it was similar in BP 0.05 and BP 0.10 (90.65%, 86.72%) compared to CON (90.19%). At the species level, the relative abundance of *Lactobacillus salivarus* was higher in BP 0.05 (40.15%) and BP 0.10 (38.58%) compared to the CON (20.04%) and PC (18.05%). A linear reduction in the weight of bursa of Fabricius (*P* = 0.022) and spleen (*P* = 0.052) was observed in birds fed graded level of BP and an increase *(P* = 0.059) in the weight of gizzard was observed in birds fed PC over BP diets. Linear and quadratic responses were observed in redness of breast muscle color in birds fed graded level of BP.

**Conclusions:**

The inclusion of the 0.05% and 0.1% BP cocktail linearly improved broiler weight during the first 7 days, 22–35 days and cumulatively, whereas 0.05% BP addition was sufficient for supporting immune organs, bursa and spleen as well as enhancing gut microbiome, indicating the efficacy of 0.05% BP as a substitute antibiotic growth promoter in broiler diets.

## Background

In response to the increase in the demand for livestock products such as meat, milk and eggs by a growing global population, livestock producers are compelled to significantly increase production of these products. Thus, large scale intensive farming systems are continuing to appear. Unfortunately, such production systems can promote disease transmission very easily due to their low genetic diversity and high stocking density, leading to concomitant production and economic losses [1, 2]. Zoonotic pathogens associated with poultry and pigs such as *Salmonella* spp., *E. coli*, *Campylobacter* spp., *Clostridium* spp., and *Listeria* spp. have been reported by European Food Safety Authority (EFSA) to be often resistant to several antibiotics [3, 4]. In this context, alternative approaches have become imperative. One option is the application of lytic bacteriophage to combat the bacterial diseases in livestock [5].

Bacteriophages are viruses that infect and use bacterial resources for their own reproduction. They are very common in all environments and have a high specificity for bacteria at infection [6]. In a review, Domingo et al. [7] suggested that bacteriophages have narrow spectrum activity against bacteria, in contrast to the broad spectrum activity of antibiotics against bacteria. Bacteriophages are specific for particular bacteria, and phage therapy is considered safe and effective in comparison to antibiotics partially because they infect one species, serotype or strain. This mechanism of action does not inhibit the proliferation of commensal intestinal flora [8, 9]. Fiorentin et al. [10] noted that the application of single oral cocktail of phages at a dosage of 10^11^ pfu decreased the occurrence of *Salmonella* *Enteritidis* strains by 3.5 log units.

In addition, other studies have also reported a successful reduction in the *Salmonella *spp. counts in chicken internal organs and excreta [11] as well as in poultry products [12, 13] with bacteriophage application. Furthermore, it has been reported that bacteriophage supplementation improved feed efficiency, liver weight and reduced pathogens in broiler chickens [14] and improved egg production and egg quality in laying hens [15].

The inclusion of phages as a feed additive may potentially provide an integrated solution to modulate the gut microbiome in chicken by reducing specific pathogenic microbial populations, thereby promoting the proliferation of beneficial microbiota, resulting in improved gut health [16].

Under bacterial challenge, bacteriophage has shown to be effective in several studies, which applied bacteriophage at different concentrations such as 0.1 mL containing 10^11^ pfu/mL, 1 mL containing 10^10^ pfu/mL or 1 mL containing 10^7^ pfu/mL respectively [17–19]. However reports on the dietary usage of a bacteriophage cocktail in birds without bacterial challenge are scarce. Thus, the objective of the current study was to assess the effects of two different concentrations of cocktail bacteriophage on the performance and production characteristics, as well as gut microbiome of broiler chickens raised under normal physiological condition (without inducing infection via bacterial challenge).

## Material and methods

### Experimental design, animals, housing and diets

Bacteriophages used in the present study was a commercial product from CJ Cheiljedang Corp. Seoul, South Korea, consisting of a mixture of phages targeting *Salmonella gallinarum*, *Salmonella typhimurium*, *S. Enteritidis, Escherichia coli* at the concentrations of 1.0 × 10^8^ pfu/g each and *Clostridium perfringens* (1.0 × 10^6^ pfu/g). A total of 504 1-day-old male broilers (ROSS 308) with the initial BW 42.9 ± 1.0 g were used in a 35-day experiment. Chicks were randomly divided into the four experimental groups, and each group had 7 replicate cages, with 18 broilers per replicate cage. The treatment groups were as follows: i) CON group (control/ basal diet without BP supplementation), ii) PC group (CON + 0.25 g antibiotics; AVILAMIX®/kg feed), iii) BP 0.05 group (CON + 0.5 g bacteriophage/kg feed), and iv) BP 0.10 group (CON + 1.0 g bacteriophage/kg feed). The bacteriophage cocktail concentrations used in the present study was based on the concentrations used in previous studies [14, 17–19] and was administrated by replacing the same amount of corn. Broiler chickens were raised in a temperature-controlled room in a three-tier stainless steel cages of identical size having 8 adjacent cages per level. The dimensions of each cage was 120 cm width × 40 cm length × 60 cm height and was equipped with 2 drinker nipples and 2 open trough feeders. Room temperature was maintained at 33 ± 1 °C for the first 3 d, and then gradually reduced by 3 °C a week until reaching 24 °C and maintained for the remainder of the experiment and the relative humidity was around 60%. The basal diet was formulated to meet or exceed all the nutrient requirements of broilers as recommended by National Research Council [20], and supplied in mash form. The experiment was divided in two nutritional phases, including starter (1 to 21 d), and finisher phase (22 to 35 d), and the ingredients and analyzed nutrient composition of the basal diet are shown in Table 1. Artificial light was provided 24 h/d by the use of fluorescent lights. All diets were fed in mash form with feed and water being provided *ad libitum* throughout the experimental period.

**Table 1:** Ingredients composition and analyzed nutrient content of basal diets (as fed-basis)

Items	Phase^a^	
	Starter	Finisher
Ingredients, %		
Corn	55.84	61.57
Soybean meal	20.50	18.67
Corn gluten meal	14.73	10.35
Wheat bran	2.00	3.00
Soybean oil	3.00	3.00
Tri-calcium phosphate	1.81	1.29
Limestone	0.94	1.13
Salt	0.46	0.41
*DL*-Methionine (98%)	0.19	0.09
*L*-Lysine (98%)	0.23	0.19
Mineral mix^b^	0.10	0.10
Vitamin mix^c^	0.10	0.10
Choline	0.10	0.10
Calculated composition		
Metabolizable energy, kcal/kg	3184	3191
Analyzed composition		
Dry matter, %	88.6	88.7
Crude protein, %	22.79	19.90
Crude fat, %	5.51	5.63
Ash,%	5.59	5.06
Ca, %	0.92	0.85
Available P, %	0.40	0.29
Lysine, %	1.06	0.98
Methionine, %	0.45	0.36

^a^ Starter diet provided during d 1 to 21; Finisher diet provided during d 22 to 35

^b^Provided per kg of complete diet: 11,025 IU vitamin A; 1103 IU vitamin D_3_; 44 IU vitamin E; 4.4 mg vitamin K; 8.3 mg riboflavin; 50 mg niacin; 4 mg thiamine; 29 mg *D*-pantothenic; 166 mg choline; 33 μg vitamin B_12_

^c^Provided per kg of complete diet:12 mg Cu (as CuSO_4_·5 H_2_O); 85 mg Zn (as ZnSO_4_); 8 mg Mn (as MnO_2_); 0.28 mg I (as KI); 0.15 mg Se (as Na_2_SeO_3_·5H_2_O)

### Sampling and measurements

#### Growth performance

Body weight and feed consumption were recorded at day 0, 7, 21 and 35. This information was then used to calculate body weight gain (BWG) average feed intake (FI), and feed conversion ratio (FCR).

#### Nutrient digestibility

Broilers were fed the respective diets containing 0.20% chromium oxide (Cr_2_O_3_) as an indigestible marker for 7 d prior to the total excreta collection period on day 35. Excreta samples were collected by placing a collecting tray under each replicate cage for the analysis of total tract apparent digestibility for dry matter (DM), gross energy (GE) and nitrogen (N). The representative feed and excreta samples were immediately stored at − 20 °C until analysis. The excreta samples were dried for 72 h at 70 °C and finely ground to allow for passage through a 1-mm screen. The procedures utilized for the determination of total tract apparent digestibility for DM, GE and N were in accordance with the methods established by the AOAC International [21]. Diets samples were analyzed for crude protein (N × 6.25; method 988.05), crude fat (954.02), ash (method 942.05), calcium (method 984.01), phosphorous (method 965.17) and amino acids (method 982.30E) following the procedures established by AOAC, International [21]. Chromium levels were determined via UV absorption spectrophotometry (UV-1201, Shimadzu, Kyoto, Japan) and the apparent total tract digestibility (ATTD) of DM, N, were calculated using indirect methods described by Williams et al. [22]. Nitrogen was determined (Kjeltec 2300 Nitrogen Analyzer, Foss Tecator AB, Hoeganaes, Sweden), and CP was calculated as N × 6.25. Amino acid analyzer (Beckman 6300, Beckman Coulter Inc., Fullerton, CA, USA) was used to measure lysine and methionine after acid hydrolysis for 24 h in HCl. Parr 6100 oxygen bomb calorimeter (Parr instrument Co., Moline, IL, USA) was used to determine gross energy by measuring the heat of combustion in the samples.

#### Excreta microbial counts

For excreta microbial counts, excreta samples were collected from all 7 replicate cages each treatment at day 35 by placing excreta collection trays under each cage. Fresh droppings (deposited within 2 h) were collected from each replicate cage per treatment and transferred into clean plastic containers. The excreta samples were immediately transferred to the laboratory in an ice box for the enumeration of *Salmonella*, *Escherichia coli* (*E. coli*), *Clostridium* spp. and *Lactobacillus*. The viable counts of bacteria in the excreta were then determined by plating serial 10-fold dilutions (in 10 g/L peptone solution) in respective media. The selective medium used for isolation of *Salmonella* was Salmonella Shigella (Difco, USA), for *E. coli*, Mac Conkey (Difco, USA), for *Clostridia* spp. Cooked Meat Medium (Oxoid, UK) and for *Lactobacillus,* Lactobacilli medium III (Medium 638, DSMZ, Braunschweig, Germany). The Lactobacilli MRS agar plates were incubated for 48 h at 39 °C, and the MacConkey agar and Salmonella Shigella agar plates were incubated for 24 h at 37 °C whereas Cooked Meat Medium agar plates were incubated at 30 °C for 24 h under anaerobic conditions. The colony counts were then enumerated and results are presented as log_10_-transformed data.

#### Ileal mucosa microbiome

For gut microbiome analysis, ileal mucosal samples were collected at day 35 from randomly selected 6 broilers per treatment groups (CON, PC, BP 0.05 and BP 0.10). Briefly, birds were sacrificed by cervical dislocation and exsanguination. After autopsy, the intestinal tract was excised and the intestinal content was removed followed by washing the intestinal segment with distilled water. Then ileal segment (distal ileum) was cut about 10–15 cm proximally to caeca and separated from the intestine and then rinsed in PBS and the mucosal layer was scraped with a glass slide. Mucosal scrapings were collected into a 50-mL conical tube and stored in an ice box and then transferred to Macrogen Inc., (Seoul, Republic of Korea) for gene sequencing. Genomic DNA extraction from the mucosal samples and the preparation of library of amplicons consisting of 16S rRNA gene and sequencing was done by Illumina MiSeq platform at Macrogen Inc. (Seoul, Republic of Korea) using MiSeq sequencing including barcoded 16S rRNA amplicons.

The 16S rRNA gene sequences were processed using the Mothur software to remove low-quality sequences [23]. Briefly, sequences that did not match the PCR primers were eliminated from de-multiplexed sequence reads. The sequences containing ambiguous base calls and sequences with a length less than 100 bp were trimmed to minimize the effects of random sequencing errors. Chimeric sequences were further deleted using the UCHIME algorithm implemented in Mothur. QIIME (Quantitative Insights into Microbial Ecology) software package (version 1.9.1) was used for de novo operational taxonomic unit (OTU) clustering with an OTU definition at an identity cutoff 97% [24]. Taxonomic assignment was performed using the naive Bayesian RDP classifier and the Greengenes reference database. Beta-diversity was measured using unweighted UniFrac distance metrics using QIIME. The unweighted UniFrace considers the community membership (presence or absence of OTUs) [25]. Principal coordinate analysis (PCoA) plots were generated based on the unweighted UniFrac distance metrics.

#### Meat quality

For physicochemical properties of the breast meat, 10 birds (*n* = 10) per treatment selected randomly at day 35 were individually weighed and killed by cervical dislocation and exsanguinated. The breast muscle (pectoralis major), bursa of Fabricius, liver, spleen, and abdominal fat were then removed and weighed. Organ weights were expressed as a relative percentage to the whole body weight. The breast muscle Hunter lightness (L*), redness (a*), and yellowness (b*) values were determined using a Minolta CR410 chromameter (Konica Minolta Sensing Inc., Osaka, Japan). The pH of the breast muscle sample was measured by a calibrated, glass-electrode pH meter (Testo 205, Testo, Germany). The water-holding capacity (WHC) was analyzed according to the methods described by Kauffman et al. [26]. Drip loss was measured using approximately 2 g of meat sample according to the plastic bag method described by Honikel [27].

### Statistical analysis

Data were analyzed using the GLM procedure of SAS (version 9.4; SAS Inst., Inc., Cary, NC, USA) in a completely randomized design. The cage served as the experimental unit for growth performance excreta microbial counts and digestibility indices whereas for microbiome and meat analysis, individual bird served as experimental unit. Pre-planned contrast was used to test the following: 1) the individual effect of CON vs. PC diets 2) the overall effect of Bacteriophage supplementation versus PC diet (PC vs. BP 0.05, BP 0.10). Furthermore, linear and quadratic polynomial contrasts were used to examine responses to supplemental graded levels of Bacteriophage at 0, 0.05% and 0.1%. Variability in the data was expressed as the standard error of means (SEM) and *P* ≤ 0.05 was considered to be statistically significant and *P* < 0.1 as trends.

For gut microbiome, analysis of similarities (ANOSIM) to determine whether the microbial compositions between the treatment and control groups were significantly different was done using QIIME software package (version 1.9.1) and was based on the unweighted UniFrac distance metrics.

## Results

### Growth performance

As shown in Table 2, the BWG tended to be higher (*P* = 0.089) in birds fed BP supplemented diets during days 1–7 and overall experiment period compared with birds fed CON diet. A significant linear effect on BWG was observed during days 1–7, 22–35, and overall experiment in birds fed the diet supplemented with graded level of BP. During days 1–7, there were no significant differences between PC and CON diet on the growth performance parameters. However, the BWG was slightly increased (*P* = 0.08) during day 8–22 in birds fed PC diet than CON diet. During days 8–22, the FI was increased (*P* = 0.017) in birds fed PC than CON diets and FI tended to be higher (*P* = 0.0796) in birds fed PC than the diet supplemented with BP. A significantly greater (*P* = 0.016) BWG and trends in increased FI (*P* = 0.06) were observed during the overall experiment period in birds fed PC diet than CON diet.

**Table 2:** The effect of bacteriophage cocktail supplementation on growth performance in broilers^a^

Items	PC	CON	BP 0.05	BP 0.10	SEM^b^	*P*-value				
		0%	0.05%	0.10%		PC vs. CON	CON vs. BP 0.05, BP 0.10	PC vs. BP 0.05, BP 0.10	Linear	Quadratic
d 1 to 7										
BWG, g	114.33	109.55	111.37	116.92	2.092	0.1235	0.0896	0.9437	0.0383	0.5101
FI, g	138.68	135.74	139.2	141.19	3.049	0.5077	0.2476	0.6833	0.1746	0.8240
FCR	1.213	1.241	1.253	1.21	0.025	0.4302	0.749	0.5538	0.2499	0.2511
d 8 to 21										
BWG, g	706.35	675.18	683.16	685.25	11.94	0.0813	0.5447	0.1471	0.6080	0.8671
FI, g	1017.19	969.53	985.43	990.12	12.93	0.0178	0.2644	0.0796	0.2770	0.7263
FCR	1.441	1.443	1.444	1.4471	0.032	0.9749	0.9422	0.9134	0.9344	0.9874
d 22 to 35										
BWG, g	977.95	927.46	958.56	982.54	20.62	0.1006	0.1052	0.773	0.0379	0.8647
FI, g	1774.22	1741.25	1744.61	1755.91	27.8	0.4127	0.4943	0.4907	0.6542	0.8881
FCR	1.820	1.883	1.826	1.791	0.044	0.3226	0.1821	0.8333	0.0840	0.7902
Overall										
BWG, g	1798.63	1712.19	1753.09	1784.71	23.00	0.016	0.0593	0.3053	0.0288	0.8576
FI, g	2930.06	2846.52	2869.25	2887.22	30.64	0.0698	0.4902	0.1842	0.2470	0.9359
FCR	1.63	1.665	1.638	1.619	0.023	0.2881	0.2052	0.902	0.1370	0.8915

^a^ Abbreviation: *CON* Basal diet without antibiotics or bacteriophage; *PC* CON + 0.025% AVILAMIX®; *BP* *0.05* CON + 0.05% Bacteriophage; *BP* *0.10* CON + 0.10% Bacteriophage

^b^ Standard error of means

Values represent the means of 7 cages with 18 chickens per replication cage

### Nutrient digestibility

The apparent total tract digestibility of DM, N and energy was comparable between CON and PC treatments. In addition, inclusion of graded level of bacteriophage to the CON diet did not affect the digestibility of nutrients in birds as shown in Table 3.

**Table 3:** The effect of bacteriophage cocktail supplementation on apparent total tract nutrient digestibility in broilers^a^

Items	PC	CON	BP 0.05	BP 0.10	SEM^b^	*P*-value				
		0%	0.05%	0.10%		PC vs. CON	CON vs. BP 0.05, BP 0.10	PC vs. BP 0.05, BP 0.10	Linear	Quadratic
d 35										
Dry matter, %	71.50	69.85	70.26	70.68	0.899	0.2104	0.5788	0.3625	0.4715	0.9976
Nitrogen, %	71.03	68.63	69.99	70.24	1.660	0.3209	0.4747	0.6592	0.4716	0.7731
Energy, %	71.76	69.92	70.55	70.86	0.918	0.1745	0.4923	0.3638	0.4338	0.8755

^a^ *Abbreviation*: *CON* Basal diet without antibiotics or bacteriophage, *PC* CON + 0.025% AVILAMIX®, *BP 0.05* CON + 0.05% Bacteriophage, *BP 0.10* CON + 0.10% Bacteriophage

^b^ Standard error of means

Values represent the means of 7 cages with 18 chickens per replication cage

### Excreta microbial enumeration

The effect of dietary bacteriophage supplementation on excreta microbiota counts in broiler chicken is presented in Table 4. The *Lactobacillus* counts were slightly increased in birds fed BP supplemented diet than the birds fed PC diet. However, the concentrations of *E. coli*, *Clostiridium perfringens*, and *Salmonella* were comparable between CON and PC group or PC and BP 0.05 and BP 0.10 groups. The excreta *Lactobacillus* counts in birds fed graded level of BP supplemented diet was not affected significantly although a numerical increase was observed.

**Table 4:** The effect of bacteriophage cocktail supplementation on excreta microbial counts in broilers^a^

Items, log_10_ cfu/mL	PC	CON	BP 0.05	BP 0.10	SEM^b^	*P*-value				
		0%	0.05%	0.10%		PC vs. CON	CON vs. BP 0.05, BP 0.10	PC vs. BP 0.05, BP 0.10	Linear	Quadratic
d 35										
*Lactobacillus*	8.93	8.99	9.115	9.206	0.096	0.6578	0.1551	0.058	0.0833	0.8632
*E. coli*	5.524	5.552	5.566	5.706	0.129	0.8789	0.5985	0.4835	0.3863	0.6798
*Clostridium perfringens*	5.512	5.601	5.543	5.529	0.142	0.6607	0.7111	0.8911	0.6689	0.8789
*Salmonella*	4.096	4.168	4.128	4.118	0.114	0.6581	0.7493	0.8478	0.733	0.9058

^a^ Abbreviation: *CON* Basal diet without antibiotics or bacteriophage; *PC* CON + 0.025% AVILAMIX®; *BP* *0.05* CON + 0.05% Bacteriophage; *BP 0.10* CON + 0.10% Bacteriophage

^b^ Standard error of means

Values represent the means of 7 cages with 18 chickens per replication cage

### Gastrointestinal microbiome

**Fig. 1:**
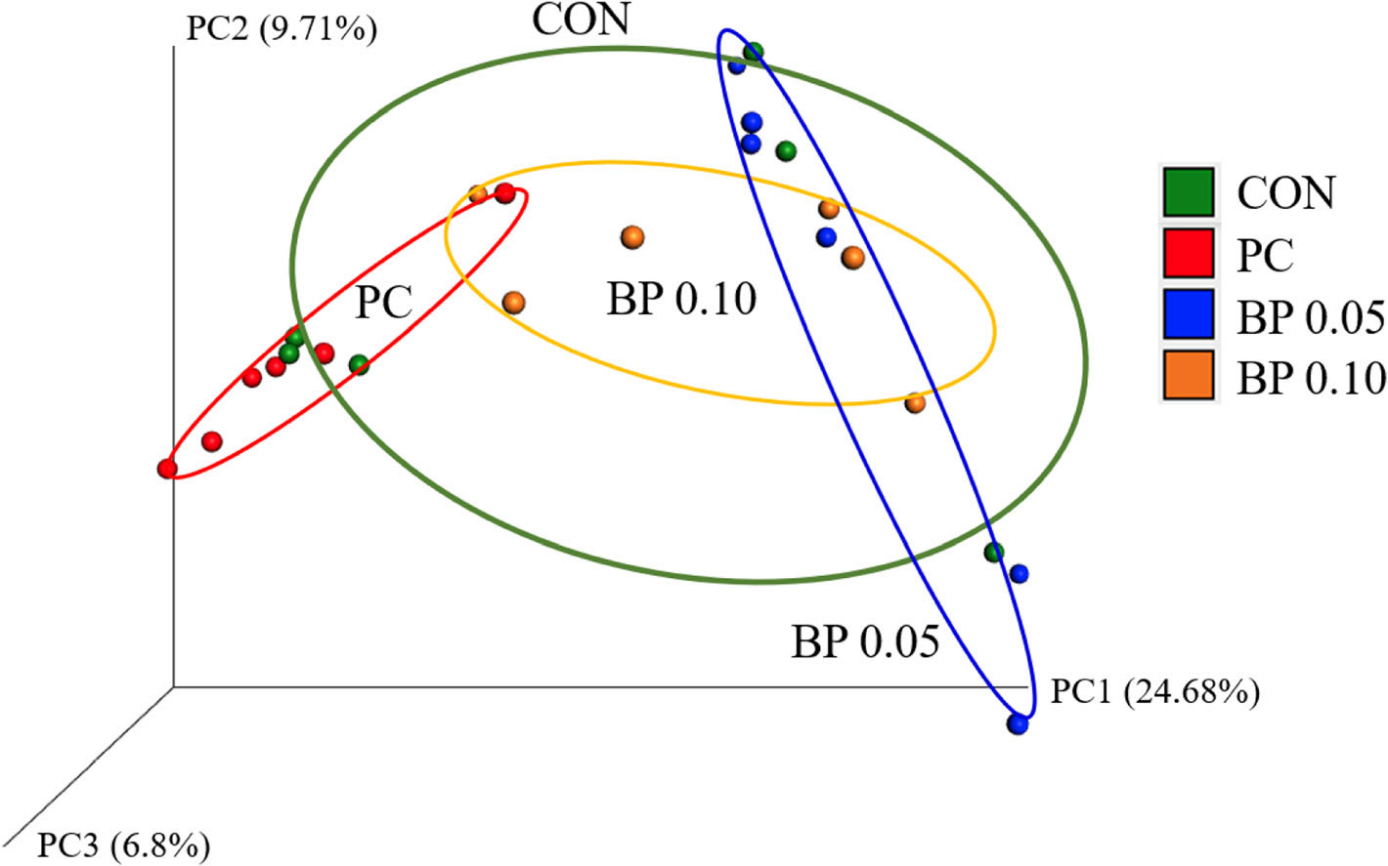
Principal coordinates analysis (PCoA) plots based on unweighted UniFrac distance metrics showing difference in microbial community structure between CON, Basal diet (green), PC, CON + 0.025% Avilamix (red), BP 0.05, CON + 0.05% Bacteriophage (blue), and BP 0.10, CON + 0.10% Bacteriophage (orange) group

To evaluate the effect of BP on the gut microbiota of broiler chicken, the mucosa-attached microbiome in the ileum were analyzed by deep sequencing. Sequencing of the 16S rRNA genes in the mucosal samples produced a total of 1,121,448 reads after quality-filtering, with a mean sequence number of 36,473 ± 37,381 reads per sample. Analysis of similarities (ANOSIM) of unweighted UniFrace distances indicated that each group was clustered significantly different excluding control group (*P* < 0.05) suggesting that microbiota of the PC and BP 0.05, BP 0.10 groups were significantly different. The unweighted UniFrac PCoA plot visually confirmed the distinct separation of microbial communities between groups (Fig. 1).

**Fig. 2:**
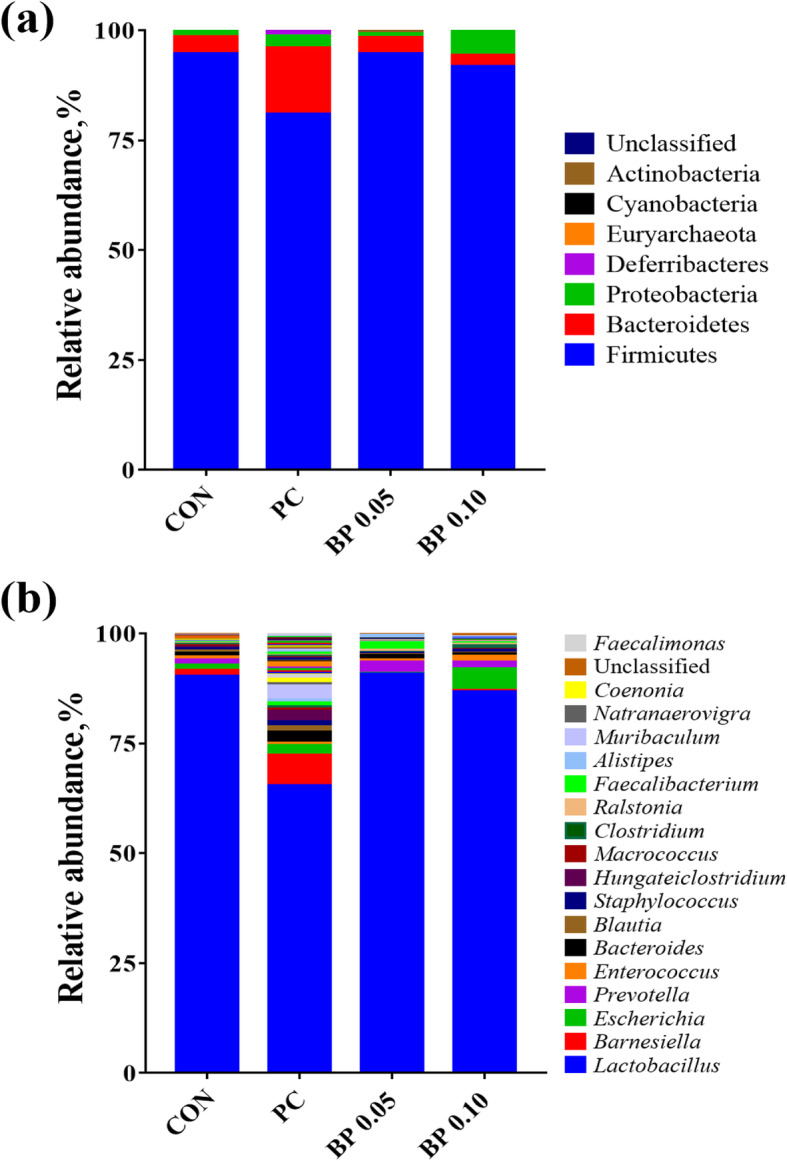
Taxonomic classification of the 16S rRNA gene sequences at the (**a**) phylum and (**b**) genus levels in the gut microbiome of broiler fed CON, Basal diet without antibiotics/bacteriophage; PC, CON + 0.025% Avilamix®; BP 0.05, CON + 0.05% Bacteriophage; BP 0.10, CON + 0.10% Bacteriophage

Comparisons of the relative abundances of the gut microbiota compositions between 4 groups at the phylum and genus levels are shown in Fig. 2. At the phylum level, the bacterial sequences from the CON samples were composed predominantly of the phyla Firmicutes (94.56%), Bacteroidetes (3.89%), Proteobacteria (1.38%) and 4 other phyla that collectively comprised 0.17% of the total sequences analyzed (Fig. 2a). PC group consisted largely of phyla Firmicutes (80.86%), Bacteroidetes (15.09%), Proteobacteria (2.78%), Deferribacteres (1.03%) and 4 other phyla which collectively comprised of 0.24% of the total sequences analyzed (Fig. 2a). In BP 0.05 group, Firmicutes (94.57%) and Bacteroidetes (3.76%) were composed as predominant, the rest 6 phyla were comprised of 1.67% of the total sequences (Fig. 2a). In BP 0.10 group, Firmicutes (91.81%), Proteobacteria (5.45%) and Bacteroidetes (2.42%) were predominant, while other 5 phyla were composed of 0.32% of the total sequences analyzed (Fig. 2a).

**Fig. 3:**
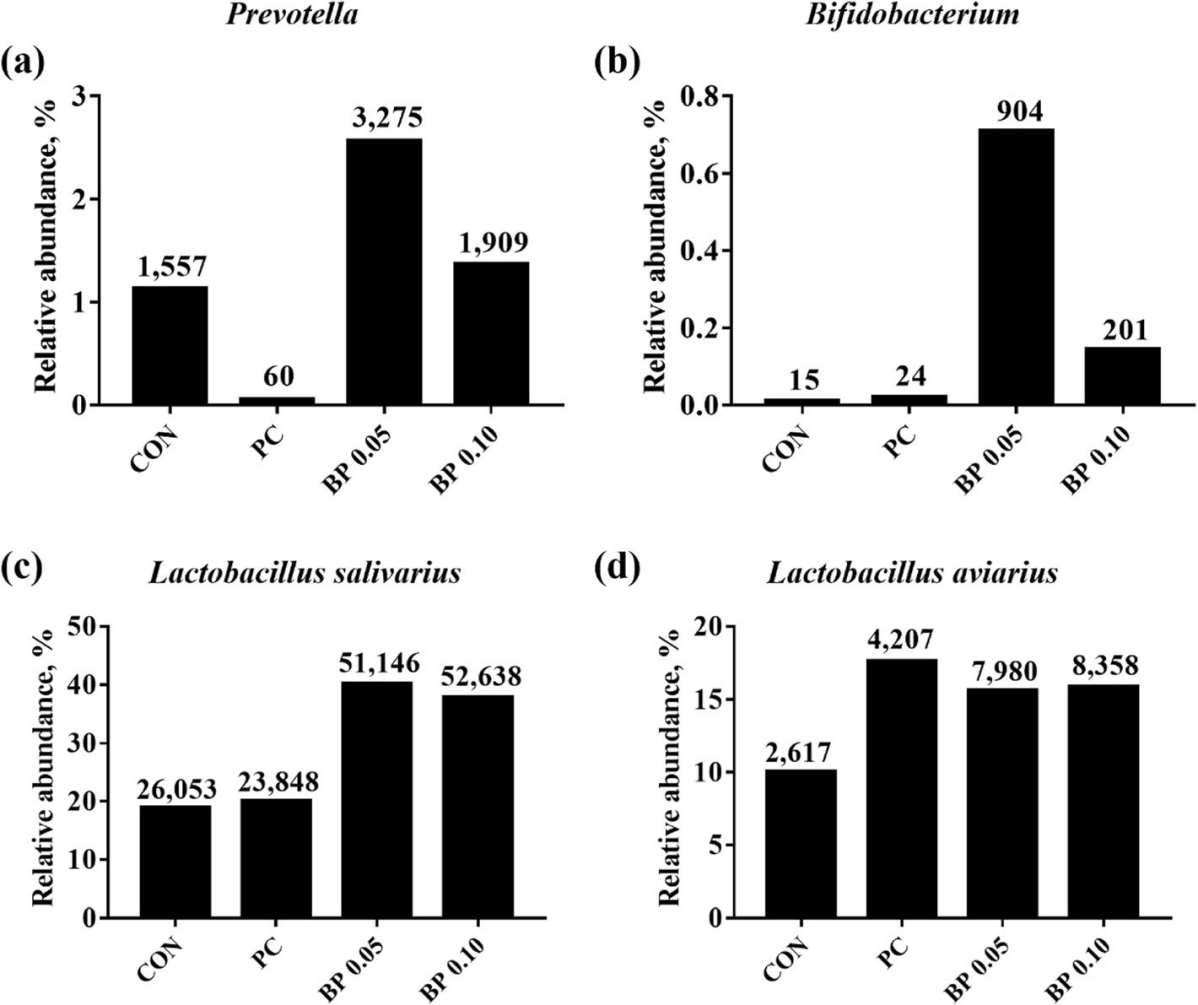
The bar plot identifying the difference in taxa between the gut microbiome of broiler fed CON, Basal diet without antibiotics or bacteriophage; PC, CON + 0.025% Avilamix®; BP 0.05, CON + 0.05% Bacteriophage; BP 0.10, CON + 0.10% Bacteriophage groups at the genus (**a**, **b**) and species (**c**, **d**) level. The numbers on each bar indicates the normalized abundance of each strains

At the genus level, *Lactobacillus* was the most enriched genera in all mucosal samples (Fig. 2b). And its relative abundance was decreased in PC (65.28%), while it was similar in BP 0.05, BP 0.1, (90.65%, 86.72%) compared to CON (90.19%). The relative abundance of *Prevotella* increased from an average of 1.15% in CON to 2.56% in BP 0.05 and 1.37% in BP 0.10 (Fig. 3a) and *Bifidobacteria* also increased from an average of 0.01% in CON to 0.70% in BP 0.05 and 0.14% in BP 0.10 (Fig. 3b).

At the species level, while *Lactobacillus salivarius* and *Lactobacillus aviarius* represented the 2 most abundant species in all groups, the relative abundance of *L*. *salivarius* increased from an average of 18.86% in CON to 40.13% in BP 0.05 and 37.80% in BP 0.10 (Fig. 3c), and the relative abundance of *L*. *aviarius* increased from an average of 10.04% in CON to 15.60% in BP 0.05 and 15.87% in BP 0.10 (Fig. 3d).

### Meat quality and organ weight

The effect of bacteriophage supplementation on organ weight and meat quality in broilers is shown in Table 5. Except for the significant reduction in relative weight of bursa of Fabricius in birds fed PC than CON diets, none of the other meat quality and organ weight parameters were affected between CON and PC diets. The relative weight of gizzard showed trends in increment in birds fed PC than BP supplemented diets. A linear reduction in weight of bursa of Fabricius (*P* = 0.026) and spleen (*P* = 0.052) relative to body weight were seen in birds fed diets supplemented with increasing level of bacteriophage. Linear and quadratic responses were observed in redness of breast muscle color for birds fed graded level of bacteriophage.

**Table 5:** The effect of bacteriophage cocktail supplementation on meat quality and organ weight in broilers^a^

Items	PC	CON	BP 0.05	BP 0.10	SEM^b^	*P*-value				
		0%	0.05%	0.10%		PC vs. CON	CON vs. BP 0.05, BP 0.10	PC vs. BP 0.05, BP 0.10	Linear	Quadratic
pH value	7.49	7.52	7.54	7.54	0.0287	0.3845	0.6189	0.1392	0.6821	0.8963
Breast muscle color										
Lightness (L*)	56.51	57.81	54.97	59.07	1.011	0.3689	0.5285	0.6803	0.3749	0.0096
Redness (a*)	12.81	13.02	14.05	11.85	0.457	0.7455	0.9000	0.8030	0.0598	0.0050
Yellowness (b*)	10.80	10.73	11.71	11.65	0.67	0.9449	0.2592	0.2932	0.3568	0.5366
WHC, %	54.79	55.26	54.69	54.97	2.465	0.8931	0.8886	0.9880	0.9279	0.8783
Drip loss, %										
d 1	3.43	3.96	3.58	3.77	0.304	0.2314	0.4583	0.5141	0.6853	0.4802
d 3	5.29	5.48	5.58	5.34	0.126	0.3022	0.9057	0.2832	0.4853	0.3250
d 5	9.17	9.74	9.65	9.16	0.374	0.2913	0.4701	0.6140	0.2868	0.6650
d 7	13.93	14.66	14.20	14.10	0.414	0.2284	0.3322	0.6660	0.3787	0.7361
Relative organ weight, %										
Breast muscle	24.28	22.97	23.46	24.07	0.592	0.1290	0.2815	0.4844	0.2076	0.9382
Liver	2.74	2.72	2.72	2.62	0.122	0.8969	0.7486	0.6388	0.5869	0.7342
Bursa of Fabricius	0.12	0.13	0.12	0.11	0.006	0.0464	0.0226	0.9944	0.0262	0.6886
Abdominal fat	1.19	1.11	1.21	1.21	0.067	0.4196	0.2395	0.7995	0.3781	0.5717
Spleen	0.18	0.19	0.18	0.17	0.006	0.1444	0.0667	0.8625	0.0521	0.8386
Gizzard	1.13	1.12	1.05	1.05	0.031	0.8796	0.0847	0.0596	0.1135	0.2432

^a^ *Abbreviation*: *CON* Basal diet without antibiotics or bacteriophage, *PC* CON + 0.025% AVILAMIX®, *BP 0.05* CON + 0.05% Bacteriophage, *BP 0.10* CON + 0.10% Bacteriophage

^b^ Standard error of means

Values represent the means of 10 chickens per treatment that are randomly selected

## Discussion

The emergence of multidrug-resistant bacterial pathogens and the imposition of ban on the usage of antimicrobials in animal production have led to a resurgence of interest in phage therapy [28]. Research on reducing zoonotic pathogens with the application of BP as a viable option in food animals has also focused on reducing the impact of infections in the animals themselves [29] thereby improving the production and performance of animals.

In the present study, a commercially available BP cocktail targeting *Salmonella gallinarum, S. typhimurium, S. Enteritidis, E. coli *and* Clostridium perfringes* was assessed for its suitability as a feed additive to enhance performance and production of broiler chickens under normal physiological conditions (without bacterial challenge).

In agreement with the findings of Kim et al. [30] who demonstrated that FI and FCR were unaffected by supplementing the broilers diet with anti-SE bacteriophage (0.05%, 0.1% and 0.2%; 10^9^ pfu/g), the inclusion of BP as feed additive at 0.05% and 0.1% levels in the present study showed no effects on FI and FCR throughout the trial, except for a trend in the linear reduction in FCR from days 22–35. However, the present study showed a significant linear increase in BWG with the increase in BP levels during the initial starter and finisher phases and overall experiment period, indicating that BP supplementation had no detrimental effect on feed consumption but promoted the BWG. In contrast, Huff et al. [31] suggested that the BWG was not affected by the inclusion of either of two bacteriophage treatments (DAF6 and SPR02), via intramuscular injection (3.7 × 10^9 ^and 9.3 × 10^9 ^pfu/mL respectively) in broiler chickens without bacterial challenge; and Wang et al. [14] noted that the supplementation of BP consisting of mixture of *Salmonella gallinarum, S. typhimurium,* and *S. Enteritidis* at a ratio of 3:3:4 at a dose level of 0.05% (containing 10^8^ pfu/g) improved FCR in  days 1 to 14 in broiler chickens but had no effect on FCR at a dose level of 0.025% (10^8^ pfu/g). In broiler production, an increase in body weight is an important parameter since lower body weight equates to an increased cost for broiler meat production [30]. The increase in BWG when BP was used as a feed additive instead of antibiotics in animal feed might be due to the inhibitive or lytic effect on harmful bacteria in the gastrointestinal tract of broiler chickens [32]. The inclusion of sub-therapeutic doses of antibiotics as positive controls in the diet of broiler chickens led to a higher BWG and FI than the birds fed a basal diet without antibiotics, which agrees with the results of several other studies [33–35], suggesting that the improvement in BWG might be due to increase in FI.

The supplementation of antibiotics or bacteriophage to the basal diet did not have significant effect on nutrient digestibility. In line with the findings of Wang et al. [14], the ATTD of nutrients was not affected by the supplementation of increasing levels of BP. Further experiments are needed to confirm the lack of response in nutrient digestibility to antibiotics or BP.

*Salmonella* is the major cause of foodborne diseases worldwide, with chickens as the main reservoir. Other zoonotic pathogens include *Clostridium, Campylobacter, E. coli*. For the control of these pathogens in poultry, bactericidal bacteriophages may provide a natural, nontoxic, feasible and non-expensive alternative. Previous works have indicated that *Salmonella* can be controlled by bacteriophages at a concentration of 1 mL containing 10^10^ pfu/mL, 0.1 mL containing 10^10 ^pfu/mL, 0.1 mL containing 10^9^ pfu/mL or 10^6^ pfu/kg [18, 36–38]. Early studies with *E. coli* also demonstrated that phage therapy at concentrations of 10^6^ pfu or 10^9^ pfu could be as efficient as antibiotics [31, 39]. The reduction in *E. coli* and *Salmonella* counts in the excreta of broiler chickens after treatment with bacteriophages has been reported [14]. Conversely, in the present study, dietary supplementation of BP did not have a significant effect on the pathogenic bacteria such as *E. coli*, *Salmonella* and *Clostridium* counts isolated from the caecal digesta. However, a non-significant linear increase in *Lactobacillus* count was observed in birds fed BP diets. The possible reason for non-significant effect of BP on nutrient digestibility and pathogenic foodborne bacterial counts among the treatments might be that the birds were raised in a hygienic environment and were not experimentally challenged with bacteria such that the gastro intestinal tract might not have been colonized by harmful microorganisms and was maintained in a healthy state.

The gastrointestinal microbiota plays a crucial role in gut associated host immune system. Moreover, the physiological development, health, and productivity are also influenced by gut microbiota. Poultry diets have a tremendous impact on the diversity and composition of the gut microbiome [40]. The manipulation of the microbial community through the inclusion of feed additives such as phage is feasible to enhance chicken growth and control either human or animal pathogens. Several studies have reported the use of bacteriophages as a feed additive in animals to control bacteria transmitted by foodstuffs. These models include the use of phages to control *Salmonella* and *Campylobacter* in broiler chickens [8, 41]. Microbiome analysis showed that Firmicutes, Bacteroidetes, and Proteobacteria are the predominant phyla in the avian gut [42], which is also supported by the results from our study. In BP 0.05 group, Firmicutes and Bacteroidetes were predominant, whereas in BP 0.10 group, in addition to Firmicutes, and Bacteroidetes, Proteobacteria were also predominant. The presence of Proteobacteria in BP 0.10 may indicate that a BP dose of 0.1% may not be favorable, as an increase in Proteobacteria may be associated with an increase in *E. coli*. In the PC group, in addition to Firmicutes, Bacteroidetes, Proteobacteria, members of phylum Deferribacteres (1.03%) were also present. Firmicutes were reduced, whereas Proteobacteria and Bacteroidetes increased in broilers receiving the PC diet compared with broilers fed the CON diet. The composition of microbiota at the genus and species level was also modified. There was a decrease in the abundance of *Lactobacillus* at the genus level in PC compared to CON, BP 0.05 and BP 0.10 treatments, and an increase in the relative abundance of *Prevotella* and *Bifidobacteria* in the phage-treated groups compared with CON and PC groups. The genus *Lactobacillus* plays a crucial role in the homeostasis of the gastrointestinal tract of metazoans [43]. At the species level, the *Lactobacillus salivarus* population in the ileum mucosa of the phage treated groups was twofold higher than in CON and PC. Shin et al. [44] noted that Proteobacteria are a possible marker of microbial instability, thus predisposing the bird to disease onset. The increase in the relative abundance of Proteobacteria and the reduction in genus *Lactobacillus* in PC suggest that antibiotic supplementation inhibited the proliferation of beneficial microorganisms in the gut. *Bifidobacteria* are used as probiotics to promote gut health, whereas *Prevotella* generate short chain fatty acid that have a specific role in the GIT, such as reducing undesirable bacterial species in the cecum and contribution to energy by gluconeogenesis [45, 46]. Therefore, the increase in relative abundance in *Bifidobacteria* and *Prevotella* in BP group (especially BP 0.05 which has higher values) in the present study, suggests the efficacy of the 0.05% phage cocktail in promoting beneficial bacteria, which would eventually contribute to improved performance and health.

Thus, the strong selective pressure exerted by lytic phages on their host communities has the potential to perturb the niche microbiota. This offers a powerful advantage over antibiotics because of their specificity, which targets only their host bacteria, suggesting a milder therapy approach towards niche microbiota. Hence, phage, when used as a substitute for antimicrobial growth promoters in animal feed, can contribute in combating the emergence of antibiotic resistance in livestock including poultry.

With regards to meat quality, a significant quadratic response in the redness and lightness values of meat color was observed with an increase in the level of bacteriophage. Although meat color is closely associated with meat pH [47], we found that the pH of breast muscle did not differ among treatments, indicating that change in color was not due to pH. In partial agreement with our findings, Wang et al. [14] demonstrated that meat pH and meat color were not affected by the addition of bacteriophage in the basal diet of broiler chickens. Besides pH, other reported factors affecting color inside the muscle include myoglobin content, muscle fiber orientation and the space between the muscle fibers [48]. Further studies on these factors with bacteriophage application could help explain the changes in color observed. With regards to organ weight, a tendency to increase the relative weight of gizzard more in PC than CON and BP groups was observed. The possible reason for the increase in the relative weight of gizzard in PC compared with CON may be the increase in FI in PC groups. The weight of spleen and bursa of Fabricius relative to the percentage of body weight in the CON group was higher than that in PC group on day 35. However, the increasing inclusion of bacteriophage to the CON diet linearly reduced the weight of spleen and bursa of Fabricius as a percentage of the body weight, indicating that, of the levels tested, BP at 0.05% was better. As the spleen and bursa are associated with immune function (as lymphoid organs) this may explain why a BP level higher than 0.05% may not be effective in improving immune function.

## Conclusions

Collectively, the data from the present study indicate that the application of bacteriophage cocktail at concentrations of 0.05% and 0.1% to the diet of commercially raised broiler chickens could linearly increase body weight gain during the first 7 days of the starter period and throughout the finisher period. Furthermore, it was observed that a 0.05% bacteriophage cocktail addition was sufficient to support the bursa and spleen which are immune organs, as well promote beneficial microorganism proliferation in the gut. These findings suggest that a 0.05% bacteriophage cocktail dietary supplementation during the finisher period would be economical and effective as a safe alternative to antibiotics for raising broilers under intensive farming systems.

## Data Availability

All data generated or analyzed during this study are available from the corresponding author on request.

## Abbreviations

ADFI: Average daily feed intake
ADG: Average daily gain
ATTD: Apparent total tract digestibility
BP: Bacteriophage
BW: Body weight
DM: Dry matter
FCR: Feed conversion ratio
GE: Gross energy
N: Nitrogen
